# Primary pulmonary leiomyosarcoma in a patient with disseminated pulmonary tuberculosis and constrictive pericarditis

**DOI:** 10.1016/j.rmcr.2026.102490

**Published:** 2026-08-27

**Authors:** Stefan Van Der Westhuizen, Allison Jane Arendse, Zane Ismail, Danie Krynauw, Dan Zaharie, Brian W. Allwood

**Affiliations:** a Division of Cardiothoracic Surgery, Department of Surgery, Stellenbosch University and Tygerberg Hospital, Cape Town, South Africa; b Department of Anatomical Pathology, Faculty of Medicine and Health Sciences, Stellenbosch University, Cape Town, South Africa; c Division of Radiology, Department of Medical Imaging, Stellenbosch University and Tygerberg Hospital, Cape Town, South Africa; d Division of Pulmonology, Department of Medicine, Stellenbosch University and Tygerberg Hospital, Cape Town, South Africa

**Keywords:** Leiomyosarcoma, Primary pulmonary sarcoma, Tuberculosis, Constrictive pericarditis, Pericardiectomy, Lobectomy

## Abstract

Primary pulmonary leiomyosarcoma (PPL) is an exceedingly rare malignancy, accounting for fewer than 0.5% of all lung tumours. Its occurrence alongside disseminated tuberculosis (TB) complicated by constrictive pericarditis has not previously been described. We report a 66-year-old HIV-negative male from rural South Africa presenting with progressive dyspnoea, found to have a right upper lobe nodule that enlarged despite adequate anti-TB therapy. Computed tomography (CT)-guided biopsy demonstrated spindle cell morphology. Cardiac MRI and right and left heart catheterisation confirmed constrictive pericarditis with severely impaired right ventricular function, precluding immediate lobectomy. A staged surgical approach was employed: anterior pericardiectomy via median sternotomy was performed first in attempt to optimise right ventricular function, followed by open right upper lobectomy. Histopathological analysis and immunohistochemistry confirmed PPL (FNLCC Grade 3, pT3N0M0) with R0 resection margins. All sampled nodal stations were free of malignancy, and whole-body positron emission tomography-CT (PET-CT) excluded nodal involvement and distant metastatic disease. The patient was discharged on postoperative day six and remained ECOG performance status 1 at 10-month follow up. This case illustrates that in TB-endemic regions, a lung nodule enlarging on adequate anti-TB therapy should prompt tissue diagnosis irrespective of an established alternative diagnosis. It further highlights the importance of a staged surgical approach when concomitant cardiac pathology compromises right ventricular function, acknowledging rapid tumour growth progression during the inter-procedural recovery interval.

## 1 Introduction

In TB-endemic regions, lung nodules are frequently encountered on chest radiographs. While tuberculomas may transiently enlarge on treatment, suggesting an encapsulated granulomatous mass, progressive growth demands tissue diagnosis to exclude malignancy [1]. PPL is the most common primary pulmonary sarcoma, yet accounts for fewer than 0.5% of all lung malignancies, with radiological features that overlap substantially with TB-related lesions and non-small cell lung carcinoma (NSCLC) [2]. The synchronous occurrence of PPL and disseminated TB with constrictive pericarditis is exceptionally rare, and this dual pathology makes the timing and sequencing of surgical management particularly challenging, especially when right ventricular function is compromised. We present this case to highlight the diagnostic pitfalls of dual pathology and the importance of staged surgical planning.

## 2 Case report

**Fig. 1:**
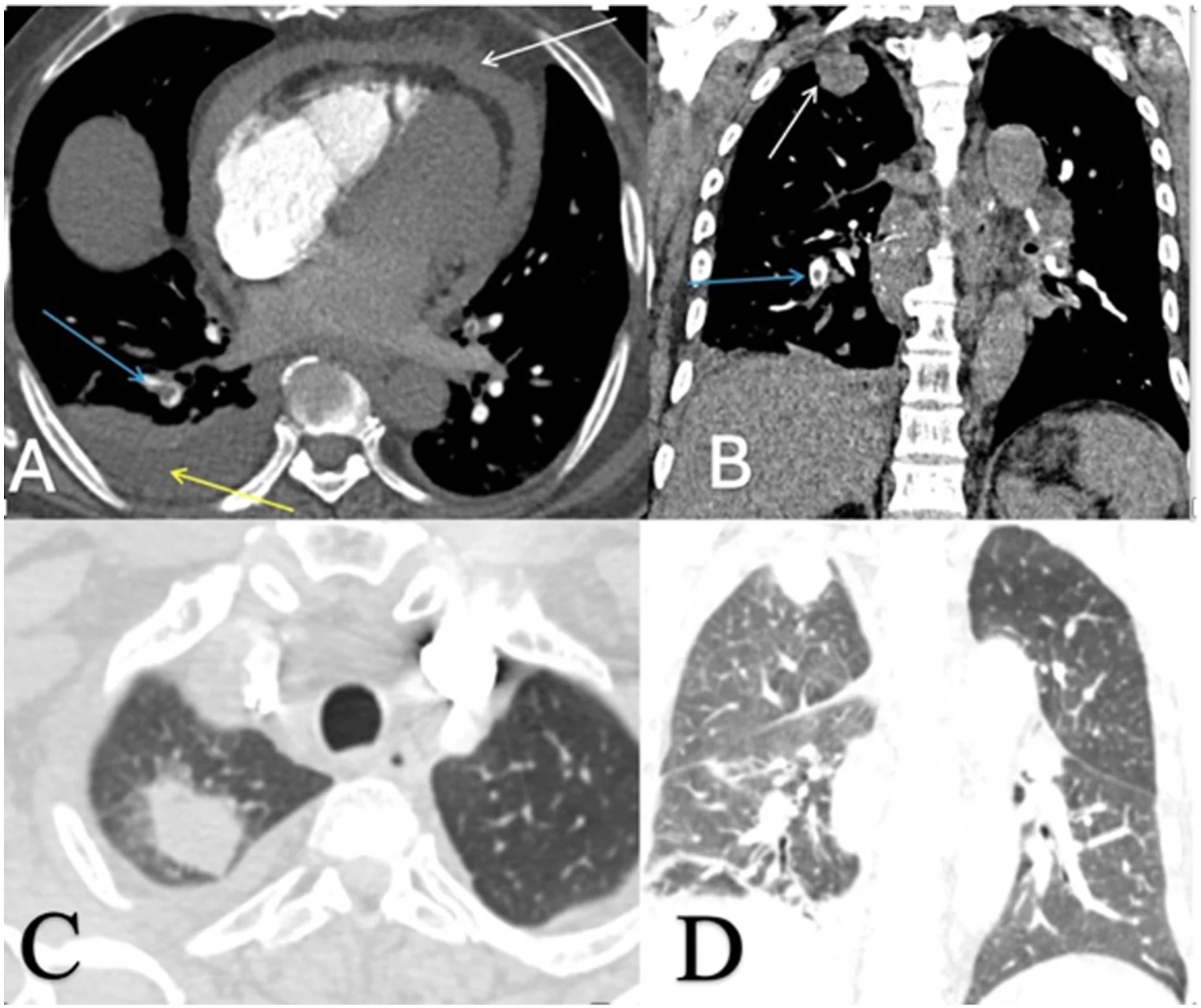
Serial imaging demonstrating disease progression 6 months apart. Initial contrast-enhanced CT (3 mm reconstructions): 1A: Axial reconstruction showing circumferential visceral and parietal pericardial thickening with associated pericardial effusion (white arrow). Right-sided pleural effusion with adjacent passive atelectasis (yellow arrow). Filling defects within the pulmonary arteries consistent with pulmonary emboli (blue arrow). 1B: Coronal reconstruction showing well-circumscribed right upper lobe apical nodule (2.1 cm × 1.4 cm × 1.3 cm) (white arrow). Calcified hilar and paratracheal lymph nodes suggesting prior granulomatous disease. 1C (axial) and 1D (coronal): 3 mm axial reconstruction clear of active pulmonary TB.

A 66-year-old HIV-negative male from rural South Africa, with hypertension and significant biomass smoke exposure, presented with a two-month history of exertional dyspnoea, orthopnoea, paroxysmal nocturnal dyspnoea, and bilateral pedal oedema with productive cough. Oxygen saturation was 91% on room air. Chest radiograph demonstrated a 3 cm right upper lobe apical nodule and a globular “water bottle” cardiac silhouette consistent with pericardial effusion. Contrast-enhanced CT confirmed circumferential pericardial thickening and effusion, the apical nodule, right pleural effusion with passive atelectasis, and hilar lymphadenopathy (Fig. 1: 1A–1D). Parenchymal features of active TB (cavitation, tree-in-bud change, or consolidation) were absent, accounting for negative sputum microscopy and TB GeneXpert. Pleurocentesis yielded an exudative effusion (adenosine deaminase 94.6 U/L); fluid PCR confirmed drug-sensitive *Mycobacterium tuberculosis*. Anti-TB therapy was commenced and the pericardial effusion was managed conservatively under cardiac MRI-guided monitoring protocol.

**Fig. 2:**
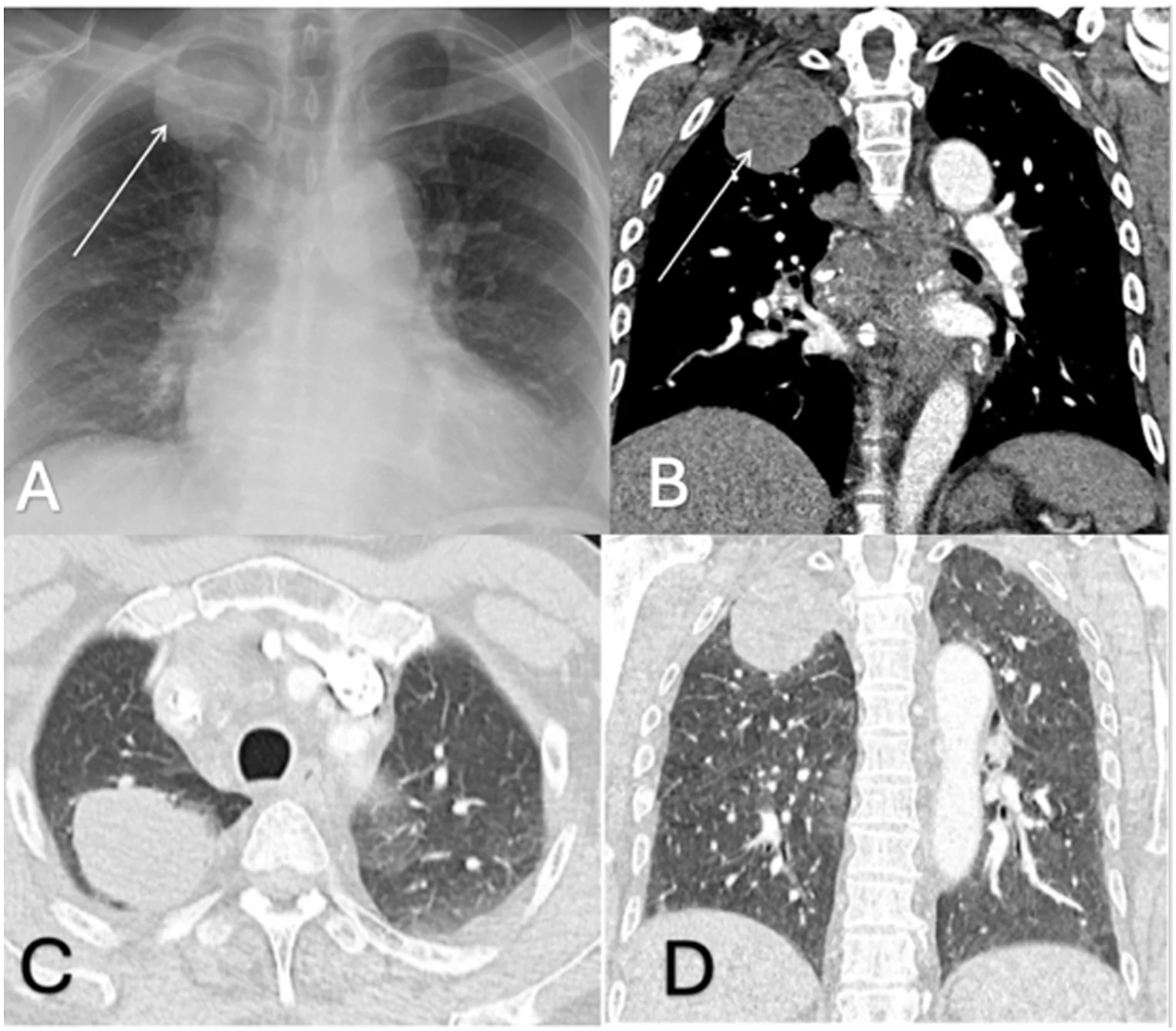
Interval imaging at six months: 2A: PA chest radiograph showing progression to right upper lobe apical mass (5.1 cm × 4.9 cm) (white arrow). Interval reduction in cardiac silhouette size. No new parenchymal features of pulmonary TB. 2B: 3 mm coronal soft tissue reconstructed contrast-enhanced CT showing right apical soft tissue mass (white arrow) with bilateral hilar and paratracheal calcified lymph nodes. 2C (axial) and 2D (coronal): 3 mm axial lung reconstructed contrast-enhanced CT showing no CT evidence of active TB.

Interval CT at six months — performed per Fleischner/SATS guideline nodule non-resolution protocol [3] — demonstrated enlargement to occupy most of the right upper lobe with mediastinal infiltration and persisting hilar lymphadenopathy (Fig. 2: 2A–2D). CT-guided transthoracic needle aspiration (TTNA) revealed spindle-shaped cells on cytology, redirecting the differential toward sarcoma. ^18^F-FDG PET-CT excluded nodal involvement and distant metastatic disease.

**Fig. 3:**
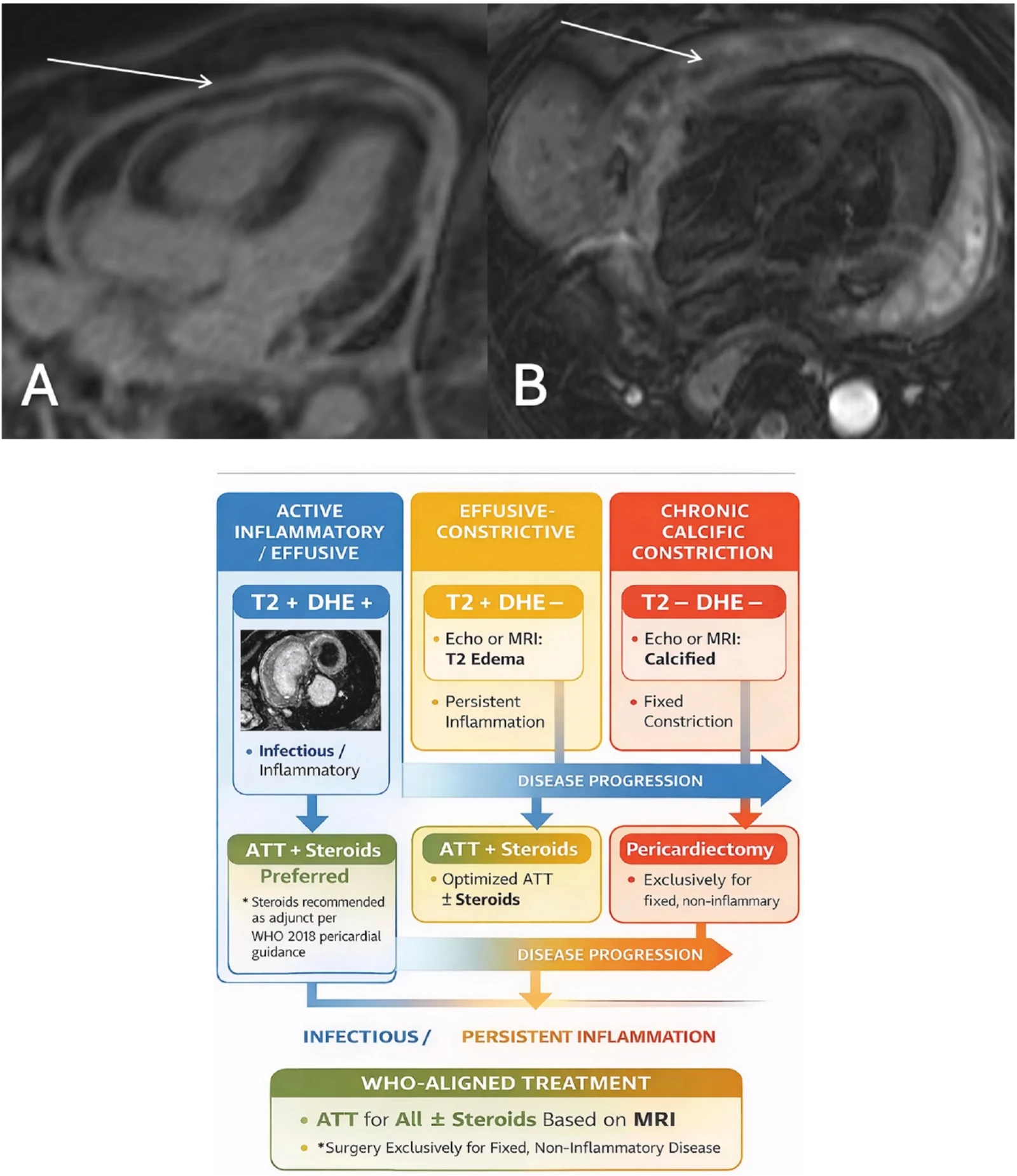
Cardiac MRI findings and the adapted flow diagram protocol. 3A: Cardiac MRI, 4-chamber view, T1-weighted late gadolinium enhancement (slice thickness: 8 mm; orientation: axial-oblique, cardiac 4-chamber plane) demonstrates circumferential thickening and enhancement of both visceral and parietal pericardium (arrow) on late gadolinium phase imaging. A complex pericardial effusion is present. 3B: Cardiac MRI, 4-chamber view, STIR sequence (slice thickness: 8 mm; orientation: axial-oblique, cardiac 4-chamber plane) shows circumferential pericardial thickening (arrow) with a large, complex pericardial effusion appearing hyperintense on STIR. 3C: Diagram adapted from Imaging-Guided Therapies for Pericardial Diseases with permission from authors.

Echocardiography demonstrated a thickened echo-bright pericardium and effusive-constrictive physiology features, severely restricted right ventricular function, and preserved left ventricular ejection fraction (55%). Repeat cardiac MRI confirmed chronic pericardial thickening with septal bounce, prompting consideration for pericardial resection. Additionally, the adapted clinical decision-making protocol used to guide clinician decision making is included in Fig. 3. Right and left heart catheterisation confirmed constrictive pericarditis with unobstructed coronaries. No features of pericardial malignancy were identified.

**Fig. 4:**
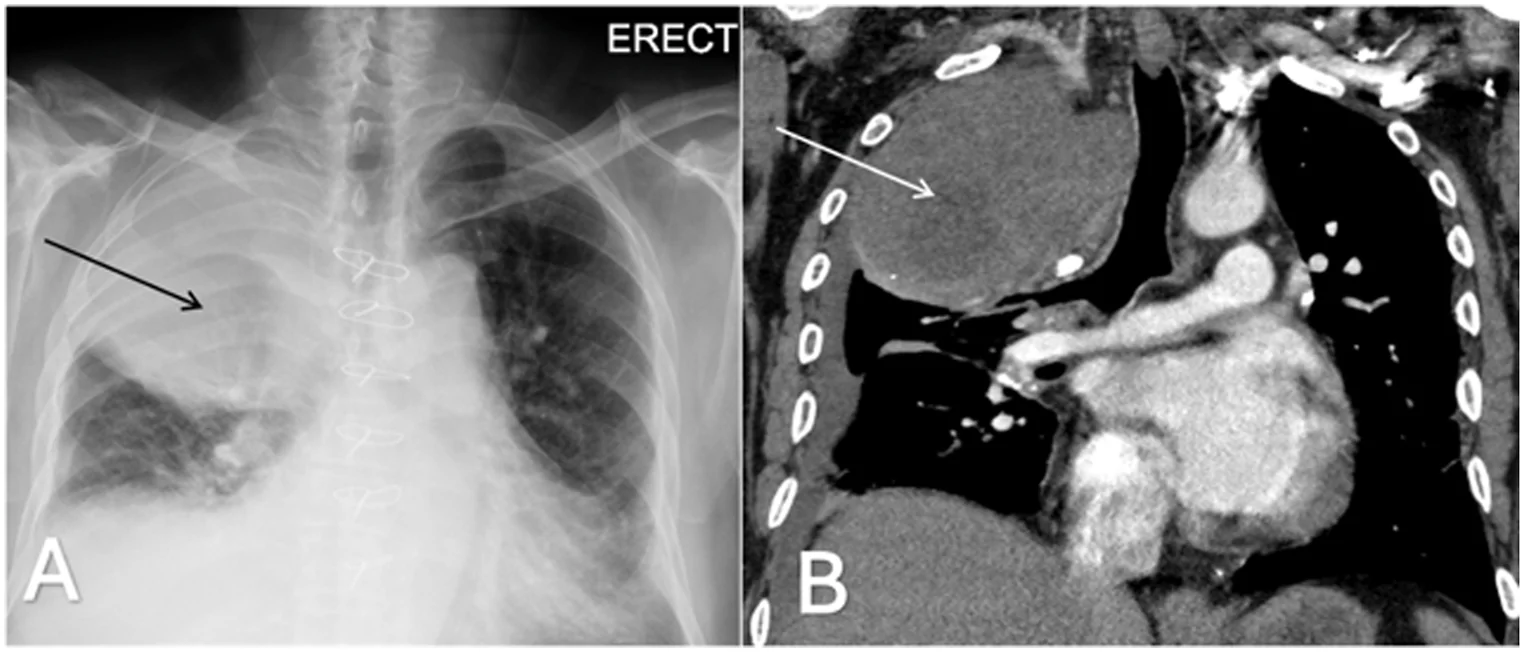
A: PA chest radiograph showing marked interval increase in size of the right upper lobe mass (black arrow). Leftward tracheal deviation indicates mass effect. Post-sternotomy wires are present following pericardiectomy. B: 3 mm coronal soft tissue reconstructed contrast-enhanced CT showing large heterogeneous right upper lobe mass with central areas of low attenuation compatible with tumour necrosis (white arrow) and tracheal deviation.

**Fig. 5:**
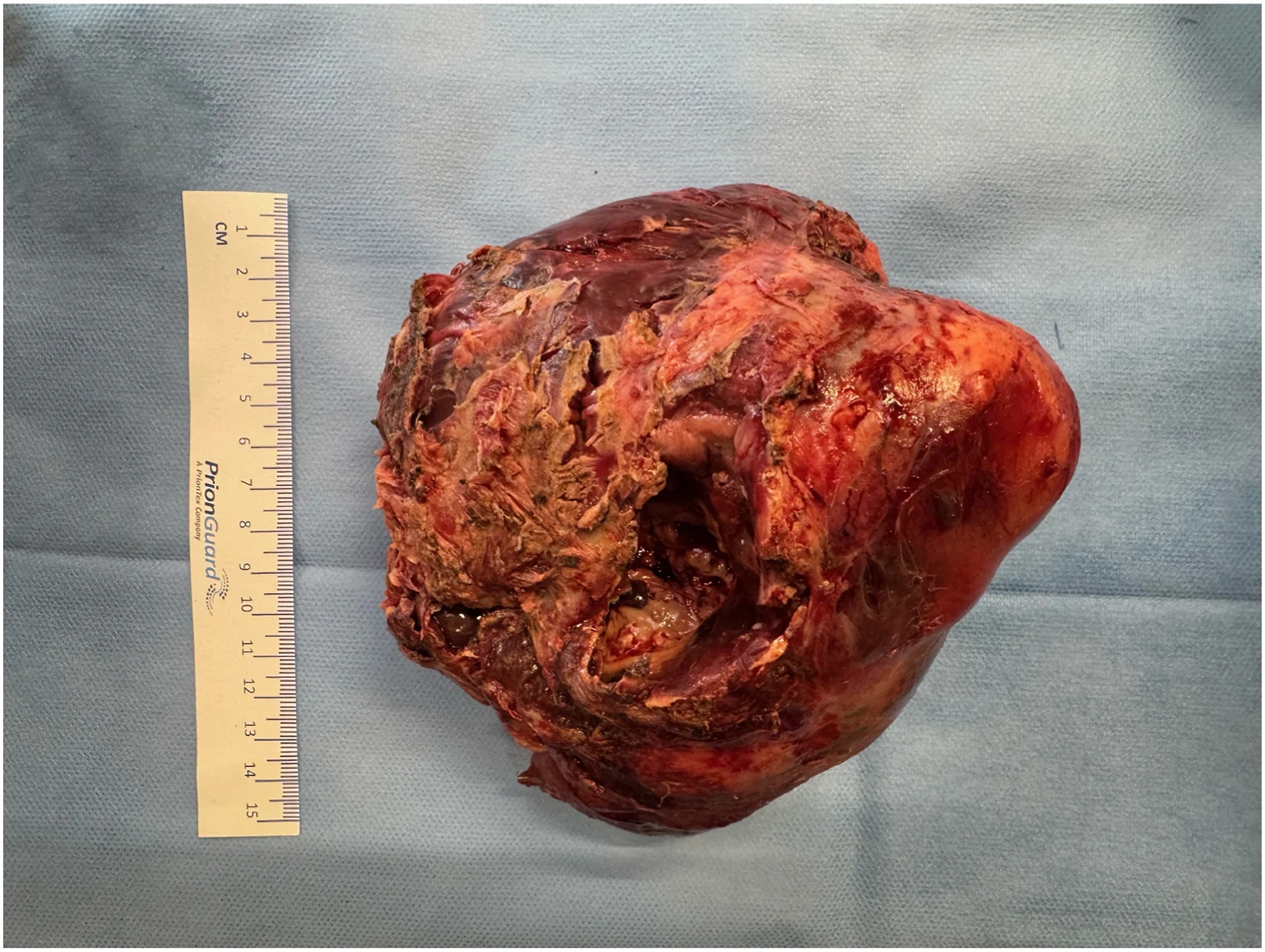
Surgical specimen following right upper lobectomy demonstrating the large (>15 cm) heterogeneous tumour mass.

The coexistence of constrictive pericarditis and suspected pulmonary malignancy created a unique surgical challenge: lobectomy in the setting of impaired right ventricular function carries substantial risk of cardiorespiratory failure, yet delay risked disease progression. Staged surgery was therefore planned. Anterior pericardiectomy via full median sternotomy was performed off-pump to optimise right ventricular function prior to lobectomy. Pericardial histology confirmed necrotic granulomatous inflammation without malignancy. On re-admission, stair-climb assessment (12 m/min) confirmed adequate cardiopulmonary reserve prior to lobectomy [4]. Rapid PPL enlargement was observed during the 6-week recovery period (Fig. 4), underscoring the need to minimise inter-procedural delay. Open right upper lobectomy with systematic hilar and mediastinal lymph node sampling followed. A large >15cm tumour was resected (Fig. 5).

**Fig. 6:**
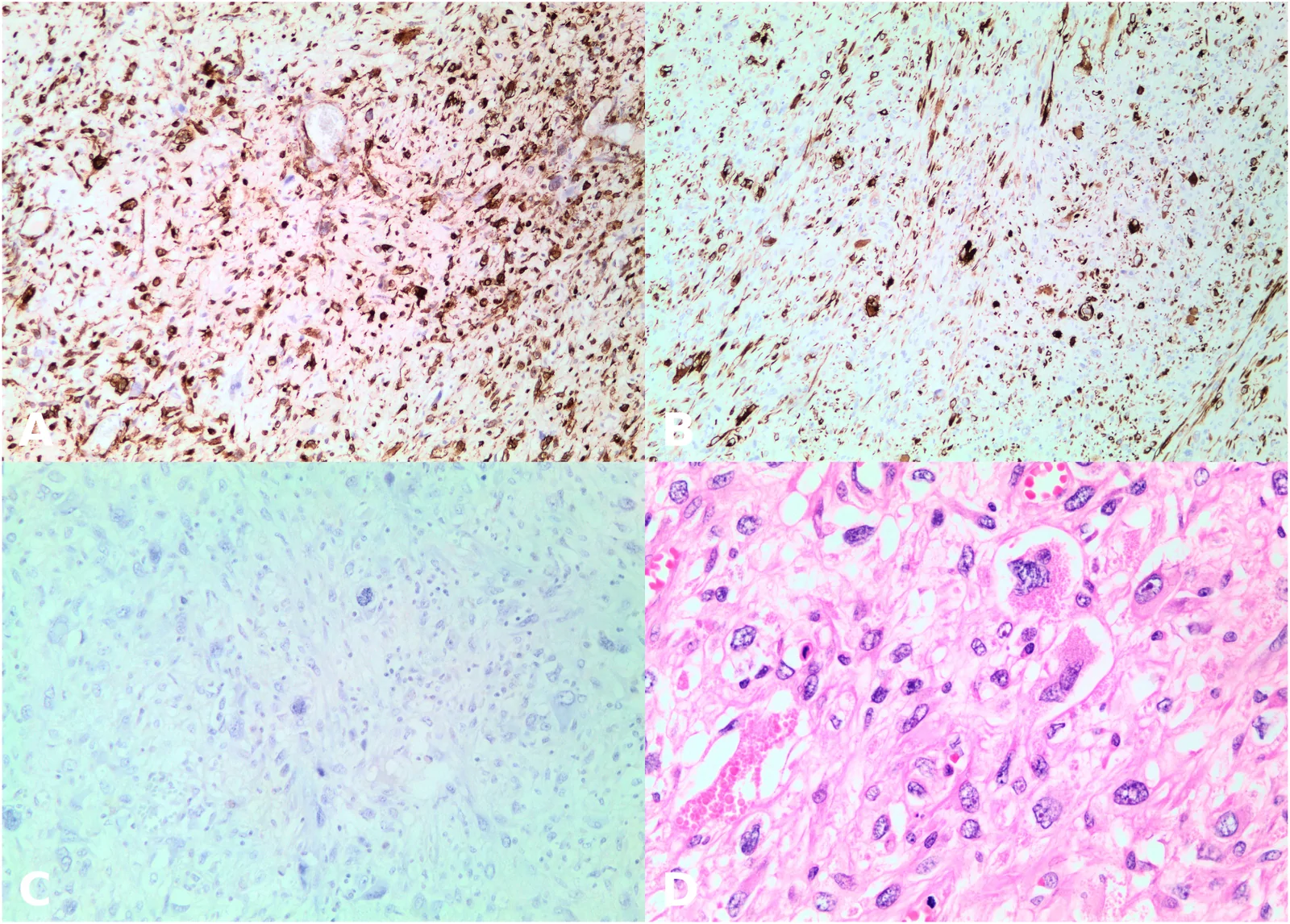
Histopathology of right upper lobectomy specimen. A: Smooth muscle actin (×100). B: Desmin (×100). C: Negative cytokeratin MNF-116 (×200). D: H&E showing highly pleomorphic spindle cells with multinucleated tumour giant cells (×400), confirming PPL.

Histological evaluation revealed a highly pleomorphic spindle cell malignancy. NSCLC was excluded by absence of epithelial markers (MNF116, CK7, CK20); neuroendocrine carcinoma (synaptophysin), melanoma (S100), and EBV-associated smooth muscle tumour (EBER) were also excluded. Positivity for desmin, smooth muscle actin, and h-caldesmon confirmed smooth muscle differentiation (Fig. 6). Grading using the Fédération Nationale des Centres de Lutte Contre le Cancer (FNLCC) system yielded a differentiation score of 3, a mitotic count of 11 per 10 high-power fields (score 2), and tumour necrosis of 25% (score 1), for a total score of 6, corresponding to a Grade 3 (high-grade) tumour. Ki-67 proliferation index was not performed, as this immunostain is not routinely available at our institution. Resection margins were R0 (closest 1.4 mm); all sampled nodal stations and pleural fluid were free of malignancy. The patient was discharged on postoperative day six and referred for adjuvant oncological assessment. At ten months, he reports ECOG performance status 1.

## 3 Discussion

Synchronous PPL and disseminated TB with constrictive pericarditis is an exceptionally rare clinical presentation. The central teaching point is that a nodule enlarging on adequate anti-TB therapy should not automatically be attributed to tuberculosis without tissue confirmation. Radiological progression despite microbiologically confirmed, drug-sensitive disease and appropriate treatment is atypical and should prompt reconsideration of the working diagnosis rather than continued observation. The relevant differential in this scenario could suggest that of a paradoxical reaction to anti-TB therapy, inadequately treated or drug-resistant infection, an alternative or coexistent infective process such as non-tuberculous mycobacterial or fungal disease, and neoplasia such as a primary lung carcinoma. As these entities cannot be reliably distinguished on imaging alone, tissue sampling of the enlarging lesion is prompted. The absence of parenchymal abnormalities on CT was consistent with TB confined to pleural and pericardial compartments, explaining negative sputum results. Spindle cell morphology on TTNA raised suspicion for a sarcoma; immunohistochemistry on the resection specimen subsequently confirmed PPL.

The staged surgical approach of pericardiectomy before lobectomy aiming to first optimise right ventricular function represents the key consideration in this case and its principal educational message. Right ventricular-pulmonary arterial uncoupling following lung resection in pre-existing right ventricular dysfunction carries significant cardiorespiratory morbidity [5]. Proceeding directly to lobectomy without first addressing constrictive pericarditis would have conferred an unacceptably high operative risk. Staged pericardiectomy preceding lobectomy was therefore essential, though this strategy itself carries risk of post-pericardiectomy right heart failure. The rapid tumour progression observed during the 6-week recovery interval emphasises the need to minimise delay wherever clinically feasible.

Establishing PPL as primary rather than metastatic requires systematic exclusion of an extrathoracic soft tissue sarcoma — in men, potential primary sites include the retroperitoneum, soft tissue, and gastrointestinal tract — in addition to thorough nodal staging [2]. In this patient, these sites were specifically evaluated on contrast-enhanced CT and whole-body ^18^F-FDG PET-CT; as no radiologically significant lesion was identified at any of them, multidisciplinary team discussion concluded that further gastrointestinal endoscopic evaluation was not indicated. Complete surgical resection remains the cornerstone of management [6]. This case demonstrates that highlights the importance of multidisciplinary decision-making in patients with synchronous rare diseases. In TB-endemic regions, a persistent or enlarging nodule should prompt tissue diagnosis regardless of any established diagnosis.

## CRediT authorship contribution statement

**Stefan Van Der Westhuizen:** Writing – review & editing, Writing – original draft, Methodology, Investigation, Data curation, Conceptualization. **Allison Jane Arendse:** Writing – review & editing, Methodology, Investigation. **Zane Ismail:** Writing – review & editing, Methodology. **Danie Krynauw:** Writing – review & editing, Visualization, Methodology, Investigation. **Dan Zaharie:** Supervision, Investigation. **Brian W. Allwood:** Writing – review & editing, Supervision.

## Patient consent statement

Written informed consent was obtained from the patient for publication of this case report and any accompanying images. Institutional ethics approval was obtained in accordance with the Declaration of Helsinki.

## Funding

This research did not receive any specific grant from funding agencies in the public, commercial, or not-for-profit sectors.

## Declaration of competing interests

The authors declare that they have no known competing financial interests or personal relationships that could have appeared to influence the work reported in this paper.
